# A Proposal for a Standardized Nomenclature of the C-arm Movements

**DOI:** 10.5435/JAAOSGlobal-D-20-00008

**Published:** 2020-02-18

**Authors:** D. Alex Stroh, Aubrey Ashie, Paul Muccino, Chelsea Bush, Daniel Kaplan, Thomas DiPasquale

**Affiliations:** From the Department of Orthopaedic Trauma, WellSpan York Hospital, York, PA.

## Abstract

No formal didactic source exists concerning terminology for movement of the C-arm in the operating room (OR). Many terminologies exist, breeding confusion among OR staff. The objective of this study was to survey the existing C-arm movement terminologies among orthopaedic surgeons and radiologic technologists and propose a standardized nomenclature moving forward.

**Methods:**

Forty-six orthopaedic surgeons and 70 radiologic technologists were surveyed. Pertinent product manuals and literature from PubMed were reviewed to find existing terms for the C-arm movement. A focus group of orthopaedic surgeons and radiologic technologists was formed and a standardized nomenclature of the C-arm terminology was developed using the Delphi method.

**Results:**

The survey response rate was 71%. The mean percentage of agreement on terms to describe movement was 47% (range, 13% to 83%). Agreement on terms to describe direction was 46% (range, 23% to 73%), and multiple frames of reference were described. No consensus was found by searching the product manuals. Using the Delphi method, we arrived at a standardized nomenclature for the C-arm movement that is reproducible and familiar.

**Discussion:**

A standardized terminology for the C-arm movement is described that will help fill a void in OR communication, combat confusion, and provide reproducible results during orthopaedic cases.

The use of fluoroscopy during orthopaedic procedures has grown over recent years. Orthopaedic utilization of fluoroscopy is increasing because of novel minimally invasive procedures, convenience, concern for patient safety, improved instrumentation, or legal necessity. By contrast, the radiologic training for orthopaedic surgeons has not advanced appreciably beyond a casual understanding of the risks associated with prolonged radiation exposure. Training for radiologic technologists, while being more standardized and thorough, rarely includes a standardized nomenclature for communication in the operating room (OR). When it does, such a nomenclature does not always align with the surgeon.^1^ It has been suggested that further education regarding the C-arm operation would be of benefit to orthopaedic surgeons in training.^2,3,4,5^

The standard C-arm has five basic independent movements that can achieve practically any projection in three-dimensional space, but these do not have standardized names. Well-intended naming conventions have been proposed in the United Kingdom, the Netherlands, and Australia but have little to no adoption, are germane only to those particular countries, or do not account for variability in the C-arm models or room setup.^6,7,8,9^ Communication errors often lead to adverse events,^10,11^ and it has been noted that communication errors regarding equipment in the OR are most common.^12^ It has also been noted that orthopaedic residents are relatively uninformed when it comes to radiologic education.^13^ The current American Board of Orthopaedic Surgeons training module for orthopaedic interns has addressed some of this knowledge deficit^14^ but does not provide a complete nomenclature. Similar training programs and modules for radiologic technologists exist, but none yet completely address these communication barriers.^15^

Owing to the lack of a complete and appropriate nomenclature, we set about to review the cardinal movements of the mobile C-arm and assess the current terminology for its use. With the aim of improving OR communication, we sought to devise an improved nomenclature for the C-arm movement that is intuitive and effective for both experienced and naive users. We hoped to provide a useful resource for orthopaedic residents and radiologic technologists during their training to become more facile with this technology. The purpose of this study was to survey orthopaedic surgeons and radiologic technologists on the terminology for the C-arm movement and to propose a standardized nomenclature to consider for adoption.

## Methods

The study design was a prospective, cross-sectional survey. Between July and December 2018, a brief electronic survey of orthopaedic surgeons (intern to attending) and licensed radiologic technologists who operate the C-arm was performed to establish what colloquial terms were used pertaining to the C-arm movement (Appendix A, http://links.lww.com/JG9/A62). Both orthopaedic surgeons and radiologic technologists were surveyed between July and December 2018. Surveys to technologists were distributed at one of their national meetings or listservs. Surveys to orthopedic surgeons were distributed to residency program directors and any orthopedic attending physicians for whom the authors had email addresses.

The survey consisted of 10 free-response questions. Each question provided three photos: the first was a photograph of the C-arm in the starting position, the remaining photos showed the C-arm and an arrow to indicate the movement of the C-arm. Survey participants were given no other direction other than to specify what they would call the movements in each picture. Survey responses were analyzed to obtain summary statistics. If specified, the name (the verb) and direction of the C-arm movement were extracted. The top five most frequently reported responses for both the movement verb and direction were tabulated. The number of top-five-reported verbs for each question that recurred in the top five verbs of other questions were tallied.

Inappropriate or nonsensical answers were counted separately but kept for analysis. This study was performed under an exempt waiver from our institutional review board, and no Health Insurance Portability and Accountability Act (HIPAA)-relevant information was used. No relevant conflicts of interest were found among the study personnel.

A search of the online and hard copy literature was performed to identify previous publications pertinent to the C-arm movement nomenclature. Product manuals were obtained from each of the main C-arm manufacturers supplying machines in the United States.

Using the responses from the survey, a focus group of orthopaedic surgeons and radiologic technologists convened to formulate a consensus on an improved, standardized nomenclature for the C-arm movements using the Delphi method. This iterative process was informed by the results of the survey, previous literature, and technical manuals from the C-arm manufacturers. Responses to each survey question were debated among the focus group, eliminating responses through discussion and review of previous literature and product manuals until a consensus was reached.

## Results

The survey was sent to 116 participants (70 radiologic technologists and 46 orthopaedic surgeons). A total of 82 responses were recorded (71% response rate, 74% for technologists, and 65% for orthopaedic surgeons). The top five tabulated responses for each survey question and percentages of respondents in agreement are shown in Table 1. No movement had perfect agreement. Focusing on only the most common response from each question, the mean percentage of agreement across questions was 47% (range, 13% to 83%) for movement verbs and 46% (range, 23% to 73%) for movement direction. Only two survey questions (Questions 8 and 10) had no overlapping verbs within the top five responses. Questions two and four had the highest number of overlap regarding verb choices (each having four of five responses recurring as a top-five response in another question). A frame of reference specifying direction regarding the patient was most commonly used, but the frame of reference/direction was inconsistently provided by the survey participants. Other frames of reference including the C-arm base, the surgeon, cardinal directions, and the OR were all used, and no single frame of reference was unique to a given movement. Given the large number of participants who did not specify the frame of reference/direction, no formal statistical analysis could be performed.

**Table 1:** Distribution of Survey Responses

Survey Question	Top 5 Responses for Verbs	No. of recurring Verbs	Top 5 Responses for Movement Direction
1	Oblique (35%)	1	Over/under (49%)
	C-over/under (22%)		Not specified (29%)
	Rainbow (12%)		Me/you (11%)
	Combo (6%)		Left/right (5%)
	Rotate (6%)		CW/CCW (1%)
2	Tilt (48%)	4	Cranial/caudal (59%)
	Move (21%)		Not specified (23%)
	Angle (11%)		Head/foot (7%)
	Cant (7%)		Superior/inferior (4%)
	Wigwag (5%)		Over/under (2%)
3	Move (62%)	1	North/south (39%)
	Slide (26%)		Proximal/distal (28%)
	Go (4%)		Head/foot (15%)
	Come (2%)		Superior/inferior (6%)
	Roll (2%)		Cephalad/caudad (5%)
4	Angle (46%)	4	Not specified (40%)
	Wigwag (23%)		Head/foot (33%)
	Invalid (11%)		Proximal distal (7%)
	Move base (2%)		North/south (5%)
	Pivot (2%)		Cranial/caudal (4%)
5	Wigwag (83%)	3	Not specified (40%)
	Angle (2%)		Head/root (30%)
	Invalid (2%)		North/south (10%)
	Oblique (1%)		Left/right (7%)
	Pivot (1%)		Proximal/distal (7%)
6	Never use It (13%)	1	Not specified (35%)
	Tilt (12%)		Cranial/caudal (23%)
	Smart view (11%)		Head/foot (13%)
	Break the C (9%)		Never use It (13%)
	Invalid (7%)		North/south (4%)
7	59% of respondents did not differentiate movements	N/A	N/A
	12% of respondents did not know this was possible		
8	Raise/Lower machine (77%)	0	N/A
	Go up/down (22%)		
9	Not specified (23%)	1	In/out (73%)
	Push/pull (20%)		Push/pull* (17%)
	Push/pull the C (17%)		Forward/backward (2%)
	Telescope (7%)		Medial/lateral (2%)
	Move the boom (6%)		Toward you/me (2%)
10	Move base (28%)	0	In/out (61%)
	Move machine (22%)		Not specified (20%)
	Not specified (18%)		Base forward/backward (7%)
	Move C-arm (10%)		Away/closer (2%)
	Move whole C-arm (7%)		Left/right (2%)

CW = clockwise, CCW = counterclockwise, N/A = not applicable.

**Figure 1:**
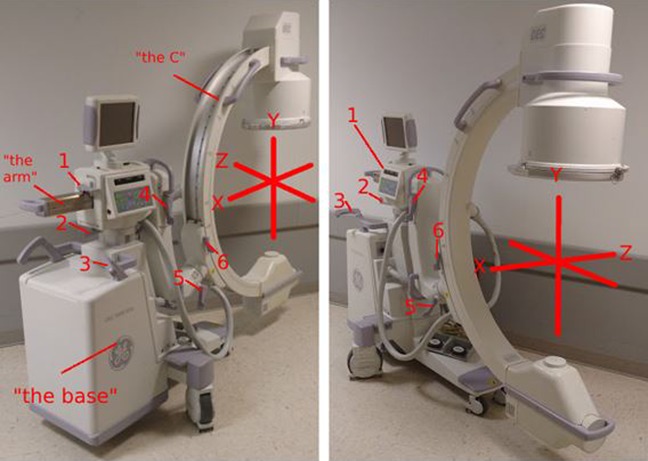
Figures illustrating the parts and coordinate axes of the C-arm: 1: arm lever; 2: wigwag lever; 3: slide lever; 4: tilt lever; 5: never lever; and 6: C lever.

Available manuals for the commonly used the modern C-arms (both standard and miniature) were found by searching online from the manufacturer websites. The nomenclature used to describe the C-arm movements is shown in Tables 2 and 3. After an iterative review of these terms and the data collected using the Delphi method, a consensus nomenclature was arrived on by a focus group of surgeons and radiologic technologists. This is shown in Table 4. As suggested in several responses, we agreed that a functional representation of the “parts” of the C-arm as shown in Figure 1 was most intuitive.

**Table 2:** Description of C-arm Movement as Specified in Company Manuals

Movement	Ziehm	OEC	Orthoscan	Hologic	Siemens	ComEd	Philips	Genoray
1	Orbital rotation	Orbital rotation	Orbital rotation	Orbital rotation	Orbital rotation	Orbital rotation	Angulation	Orbital rotation
2	Angulation	Lateral rotation	Pivot rotation	Pivot rotation	Angulation	Pivot rotation	Rotation	CW/Anti-CW rotation
3	N/A	N/A	N/A	N/A	N/A	N/A	N/A	N/A
4	Swivel/panel	Wigwag	Lateral rotation and wigwag	Panning	N/A	Panning motion	Panning	Panning
5	Swivel/panel	Wigwag	Lateral rotation and wigwag	Panning	N/A	Panning motion	Panning	Panning
6	N/A	L-arm	N/A	N/A	N/A	N/A	N/A	N/A
7	N/A	Flip-flop	N/A	N/A	N/A	Reverse position	N/A	N/A
8	Vertical movement	Vertical travel	Vertical range	Vertical travel	Vertical travel	Vertical travel	Height movement	Up/down movement
9	Horizontal movement	Horizontal travel	N/A	Horizontal travel	Horizontal travel	Horizontal travel	Displacement	Horizontal movement
10	N/A	N/A	N/A	N/A	N/A	N/A	N/A	N/A

CW = clockwise, OEC = OEC Medical Systems, Inc., N/A = not applicable.

**Table 3:** Description of the C-arm Movement as Specified in the Previous Literature

Movement	Chaganti et al^7^	Williams et al^8^	Pally et al^17^	Yeo et al^6^	De Muinck et al^9^
1	Orbital rotation	Rollover/under	Rotate over/back	Swing up/down	Can’t forward/backward
2	Angulation	Tilt north/south (or left/right if working on upper extremity)	Tilt distal/proximal	Roll up/down	Can’t toward the foot-end/head-end
3	N/A	Move north/South (or left/Right if working on upper extremity)	(Verb not specified) proximal/distal	Base up/down	C-arm to the head-end/foot-end
4	Swivel/panel	Swing north/South” (or left/Right if working on upper extremity	Swing proximal/distal	N/A	Turn to the foot-end/head-end
5	Swivel/panel	N/A	N/A	Rock up/down	N/A
6	N/A	N/A	N/A	N/A	N/A
7	N/A	N/A	N/A	N/A	N/A
8	Vertical movement	Raise up/toward ceiling, lower down/toward floor	Raise/lower		C-arm up/down
9	Horizontal movement	N/A	N/A	Arm in/out	N/A
10	N/A	Come in/back out	(Verb not specified) in/out	N/A	Drive in/out

N/A = not applicable.

**Table 4:** The C-arm Movement Terminology Based on the Results of the Present Study

Movement	Description/Terminology
1	C-over/under
2	Tilt towards the patient's _________(head/foot/fingers/chest)
3	Slide your base to the patient's __________(head/foot/fingers/chest)
4	Angle your base towards the patient's ___________ (head/foot/fingers/chest)
5	Wigwag towards the patient's ________(head/foot/fingers/chest)
6	^a^Rotate through the never-lever towards the patient's __________(head/foot/fingers/chest)
7	Flip 180 through the tilt-lever vs. the never-lever
8	Raise/lower your arm
9	Push your arm in/Pull your arm out
10	Slide your base in/out

a We suggest avoiding use of this lever in routine cases.

## Discussion

Our results demonstrate a wide array of responses to the movements of the C-arm. The nomenclature we devised is familiar—terms used were proposed within the survey, judged to have little overlap and good consistency, and were germane to both surgeons and radiologic technologists.

In a three axis “XYZ” coordinate model (Figure 1), there are six independent movements: translational movement along X, Y, and Z and rotational movement around X, Y, and Z. The C-arm performs all translations, two rotations about the X- or Z-axis at the center of the “C,” and imperfect rotation about the Y-axis (centered at the base, rather than within the “C”). Owing to this, rotation about the Y-axis will always produce translation along the XZ plane. Some C-arm models also have an additional joint located at a bend in the “arm” of the C-arm, termed the “L-arm” by the manufacturer. This lever (the “SmartLock,” “Never Lever,” “bottom lock”, etc.) unlocks simultaneous movements about an axis 45° from both the X-axis and Y-axis, which is difficult to interpret because it is accompanied by unintended changes to our typical anterior-posterior and lateral views. We discourage the use of this lever during routine imaging and prefer to name it the “Never Lever.” Its use is in circumstances when the required rotation about the Z-axis is beyond the baseline range of the C-arm. The L-arm can be “broken” to allow more “C-over/under” as required (eg, Judet views or combined iliac/obturator outlet/inlet). In translation, the C-arm is only restricted to single axis movement along the Y-axis while angling the “Slide Lever” anywhere between 0° and 90° allows movement in the XZ plane.

Previous works provide context for the current study. Trehan and Tennent first reported on the results of a survey of the C-arm movement (32 radiologic technologists and 48 surgeons) in 2006.^16^ They reviewed the translational movements and found subjective reductions in OR miscommunication. Chaganti et al^7^ surveyed 100 Orthopaedic surgeons and radiologic technologists on their familiarity with the C-arm nomenclature based on the Ziehm product manual. They found good agreement on the translational movements, but little agreement of how to specify angular or rotational movements. Importantly, this survey did not include a C-arm model with the added functionality imparted by the “Never Lever.” Williams et al^8^ showed a significant reduction in time and radiation dose during the C-arm targeting maneuvers when a standardized nomenclature was used. Their simple and effective nomenclature accounted for differences between a lower (C-arm perpendicular to table) and upper extremity operation (C-arm parallel to the table). The added confusion of the “Never Lever” was not explored. This nomenclature references the radiologic technologist's point of view, which adds extra effort for surgeons thinking in an entirely different frame of reference. Pally and Kreder^17^ surveyed Canadian orthopaedic surgeons and radiation technologists to develop their own C-arm nomenclature. Many of their terms are reproduced in our survey; however, their nomenclature relies on the C-arm coming in perpendicular to the long axis of the OR table. Yeo et al^6^ showed a reduction in time and number of exposures during a C-arm simulation when using a nomenclature of their design. De Muinck Keizer et al^9^ both formed a nomenclature based on their own survey and tested its effectiveness with a simulation task. They found a reduction in time, number of images, and radiation dose during their simulation when using the nomenclature. This Dutch nomenclature has sensible English translations; however, many of them are not in typical use based on the results of our survey. They also advocate for the use of hand gestures, which we discourage.

Several important limitations should be noted regarding the development of the consensus nomenclature. First, responses that may have achieved high agreement in a given movement (even a majority) were nevertheless rejected if they had substantial (>1%) overlap with another movement. We discourage hand gestures, often precluded by the need to hold a reduction. These were subjective decisions. Finally, no nomenclature can be complete because there will always be instances where it is more intuitive to ask that a certain anatomic feature be “centered” or that the C-arm be moved to avoid impinging on the patient.

In conclusion,an optimal nomenclature is elusive, but this standardized nomenclature is an improvement. A trauma surgeon may ask for “inlet” and “outlet,” but this does not translate to the upper extremity, some spine surgeons know it as “Ferguson tilt,” and there are other terms used outside of orthopaedics. A reasonable next step would be to expand this exercise to other C-arm heavy specialties (interventional radiology, cardiology, gastroenterologists, etc.) to further develop this nomenclature. Fluoroscopic exposure should be as low as reasonably achievable. In the era of standardized education for orthopaedic surgeons and radiation technologists, we have the opportunity to instill a common nomenclature with the end goal to reduce miscommunication, avoid unnecessary radiation, and improve the OR environment for all who have to work in it.
